# Deep learning-enabled morphology analysis of bovine sperm for label-free imaging flow cytometry

**DOI:** 10.3389/fvets.2026.1634224

**Published:** 2026-05-08

**Authors:** Anel Umirbaeva, Andrey Kurenkov, Bolat Seisenov, Kanat Zhumanov, Kadyrbay Tadziyev, Mirzhan Mustafin, Ivan A. Vorobjev, Natasha S. Barteneva

**Affiliations:** 1 Department of Biology, School of Sciences and Humanities, Nazarbayev University, Astana, Kazakhstan; 2 Department of Computer Sciences, School of Engineering and Digital Sciences, Nazarbayev University, Astana, Kazakhstan; 3 JSC Republican Center of Breeding in Livestock Asyl-Tulik, Kosshi, Kazakhstan; 4 National Laboratory of Astana, Nazarbayev University, Astana, Kazakhstan; 5 The Environmental Research and Efficiency Cluster, Nazarbayev University, Astana, Kazakhstan

**Keywords:** artificial intelligence, bovine sperm, cryopreservation, deep learning, imaging flow cytometry, label-free imaging, sperm morphology

## Abstract

Data analysis of sperm morphology is critical for evaluating bull fertility, yet it is commonly performed using light microscopy and staining techniques in a subjective and manual manner. In this study, we introduce a scalable, high-resolution approach combining label-free Imaging Flow Cytometry with deep learning for automated classification of bovine sperm morphology. We analyzed 401,535 single-cell images obtained out 1.8 million events acquired at 40 × magnification from three bull breeds - Kazakh Whitehead, Auliekol, and Simmental, from fresh and cryopreserved sperm - providing a uniquely large and diverse dataset. The dataset was used for training and evaluation of deep learning models. Multiple classification strategies and architectures were evaluated using consistent training and data preparation strategies, including MobileNetV3-Large, EfficientNetV2-S, ResNet-50, and ConvNeXt-Tiny. Among these, the ConvNeXt-Tiny yielded superior results, achieving an accuracy 91.1% and a macro F1-score of 0.91 after training with a Linear Probing and Fine-Tuning (LP-FT) strategy, and was chosen as the primary model to classify spermatozoa into eight distinct morphological categories. Testing across different conditions and breeds resulted in a 10–15% drop in generalization performance, highlighting the limitations in generalization due to dataset variability and underscoring the need for larger, standardized imaging protocols. The proportion of morphologically abnormal spermatozoa differed by seasons and following cryopreservation. This study highlights the advantages of integrating IFC and artificial intelligence algorithms for robust, high-throughput, and objective label-free assessment of spermatozoa morphology in both fresh and cryopreserved sperm, offering a promising tool for improving fertility diagnostics and breeding strategies in veterinary practice.

## 1 Introduction

The success of fertilization relies on high sperm quality, assessed through various indicators, including sperm cell count, motility, morphological structure, and overall functionality (1–4). Accurate, multi-parametric evaluation of these quality indicators is critical for the efficiency of assisted reproduction technologies (5, 6). Traditionally, the routine evaluation of semen has involved the assessment of morphological abnormalities (7–9); however, this process remains challenging and primarily relies on staining techniques (10, 11). These difficulties are particularly significant in sperm cryopreservation and refrigeration techniques in livestock, which alter the viability, motility, morphological and functional characteristics of sperm, emphasizing the need for accurate and predictive assessment methods (12–15). The ability to cryopreserve livestock sperm has existed for over 80 years, yet many of the techniques used today remain empirical, with only a few improvements made to the procedure.

Even with the seemingly widespread success of cryopreservation and sperm banking, it is not completely understood how to improve a cryosurvival of spermatozoa. Cryodamage can occur due to various factors, such as damage to the plasma membrane (16, 17), DNA integrity (18, 19) and sperm chromatin (20, 21), ionic and osmotic imbalances, and the activation of proteases during freeze–thaw process (22–24). Additionally, protein denaturation can be involved in apoptotic changes and affect the acrosome and flagellar structures (25–29). Reactive oxygen species (ROS) (30–34) and reactive nitrogen species (RNS) (28, 35–37), the expression of antioxidant enzymes, and levels of mitochondrial membrane potential (MMP) and ATP are all critical in the cryosurvival of sperm (29). The increased percentage of sperm abnormalities after freezing include coiled and bent spermatozoa tails (38, 39); cracked tails and detached heads (20). Moreover, a subpopulation of sperm could be induced partially or totally to initiate the ‘cryo-capacitation’ process (40). The most pronounced damage happens in the sperm plasma membrane, likely affecting CatSper voltage and pH-sensitive channel functionality (28, 41–46), and outer acrosomal membrane after the freeze-thawing step (47, 48).

Morphological abnormalities are often associated with other biomarkers of sperm function (49–51). For instance, defects in the sperm head have been linked to the altered expression of proteins involved in sperm capacitation and sperm-egg interaction (50), and chromatin packaging anomalies that may compromise nuclear membrane integrity (51). Tail abnormalities are frequently associated with impaired motility and mitochondrial dysfunction (52, 53), and severe flagellar deformities can result in complete loss of sperm motility and infertility. Other morphological abnormalities, such as excess residual cytoplasm (54), may indicate incomplete maturation of cells or high osmotic stress during storage or transportation, ultimately reducing viability (55).

Imaging flow cytometry (IFC) is one of the few technologies capable of simultaneously analyzing multiple functional parameters, coupled with morphological assessment, at the single cell level (56–58). Considering the complex structure of spermatozoa and the frequent occurrence of multiple co-existing abnormalities within a single cell, traditional manual morphological assessment, which is a long-standing gold standard for identifying structural anomalies (59), has significant limitations when it comes to accurate categorization of images.

Moreover, IFC enables simultaneous multiparametric functional analysis of tens of thousands of images at the single-cell level (56–60). Biomarker discovery efforts in animal and human sperm selection are also closely related to the adaptation of conventional and imaging flow cytometry (FC and IFC) (61–65). Artificial intelligence (AI) algorithms, particularly convolutional neural networks (CNNs), have demonstrated a strong performance across biomedical image analysis and are well-suited to learning discriminative features for sperm quality assessment and selection (66–75). IFC is, thus, a suitable option for the high-throughput sperm analysis, resulting in tens of thousands of label-free single-cell events with about a thousand morphological parameters. Such large image datasets allow training of the AI models for faster and more precise sorting into morphological categories (70).

While previously sperm morphological analysis was limited to the classification of specific cell regions, such as head shape or tail anomalies (71, 72), novel trends are focusing on live label-free whole-cell classification (73–75). Integration of CNNs with image-based flow cytometry has allowed quantification of boar semen morphology and label-free acrosome status evaluation at high throughput using IFC, achieving high F1 scores across magnifications (49). In parallel, stain-free optofluidic time-stretch imaging flow cytometry paired with CNN-based segmentation has enabled high-throughput label-free morphological assessment, underlining the suitability of CNN-IFC pipelines for objective, unstained, whole-cell semen evaluation (76). Still, most researchers utilize microscopy-based image datasets, stained, and within-domain evaluation, with limitations arising due to the large amount of data required for training a machine learning algorithm (77). Our study aims to address these gaps, particularly in the comparison of fresh-frozen sperm, cross-breed generalization, and limited sample-efficiency analysis.

The primary goal of this research was to quantitatively profile a large, label-free IFC database and analyze CNN robustness across classes, conditions, and breeds by creating a dataset of fresh and frozen semen from three local bull breeds - Kazakh Whitehead, Simmental, and Auliekol - and by evaluating domain shift (fresh-frozen, breed to breed). The findings from these analyses offer valuable insights into the potential application of this model in biological and veterinary contexts.

## 2 Materials and methods

### 2.1 Animals and semen collection

Sperm from six beef bull sires (4 Kazakh Whitehead, 1 Simmental, and 1 Auliekol) were used in this study. All bulls were reared with similar management conditions on a diet of mixed grass hay and drone homogenate in the Republican Center “Asyl-Tulik” for breeding in livestock. For this study, two fresh ejaculates of each bull (spring and fall seasons) were used. Cryopreserved straws of 6 qualified ejaculates (motility > 60%) of bulls obtained during the spring–summer season and diluted in OptiXcell® (IMV Technologies, France) in a 1:1 ratio with the final concentration app. 0.5 billion cells/ml in a 0.25 mL straw were also used for this study. The final concentration of both frozen and fresh sperm samples was app. 250 mln cells/ml. The sperm were not collected specifically for research but were a byproduct of the standard production process and, therefore, exempt from Nazarbayev University Institutional Animal Care and Use Committee (IACUC) oversight. All procedures in the Republican “Asyl-Tulik” facility were performed in accordance with relevant animal commercial production procedural guidelines to ensure animal welfare and conformance to ethical integrity regulations. None of the animal procedures were carried out in Nazarbayev University.

### 2.2 CASA analysis of motility

Computer-assisted sperm analysis, or CASA, was performed by the Hamilton Thorne Motility Analyzer HTM-2030 (Hamilton Thorne LTD., USA) to evaluate sperm motility values, including cell count, progressive motility (PM, %), total motility (TM, %), elongation (μm), mean average path velocity (VAP, μm/s), and other motility parameters. For each sample, on average, 100 spermatozoa were analyzed in five fields of view. Sperm concentration was measured using a photometer FEK-M (HV-Lab, RF) instrument. Results of CASA analysis for each of the ejaculates obtained are given in Supplementary Table S1. Each ejaculate sample of fresh semen for all bulls was used for both CASA analysis and IFC analysis for viability assessment with propidium iodide (Sigma-Aldrich, USA) staining. Frozen samples, taken as 0.25 mL straws, were thawed at 37 °C, and then analyzed under IFC for both viability and morphological assessments.

### 2.3 Imaging flow cytometry: data acquisition

For each of the six bulls, there were two fresh semen ejaculates obtained during the spring and fall periods and one frozen ejaculate obtained in summer. The spermatozoa images were acquired using ImageStreamX Mark II (Amnis-Cytek, USA) imaging flow cytometer, equipped with 405, 488, 561, and 642 nm lasers and a brightfield light source (Amnis-Cytek, USA) with a 40x objective. IFC data were initially analyzed using IDEAS vs.6.2 software (Amnis-Cytek, USA). Two files with 50,000 events were recorded for each bull semen sample, and from the initial 1.8 million images, severely blurred, truncated, and debris images were removed through gating. The average number of extracted images was about 25,000 for each sample from both groups, as given in Supplementary Table S2. Finally, we applied center cropping and filtering, discarding images with a shorter edge <50 pixels. This threshold was chosen because frames at or below this scale were mostly debris or lacked discernible morphological cues that could ensure quality labeling. The resulting set after filtering out debri contained 401,535 images (*n* = 257,844 for fresh and *n* = 143,691 for frozen) and was used in all downstream steps.

### 2.4 Morphology assessment

**Figure 1:**
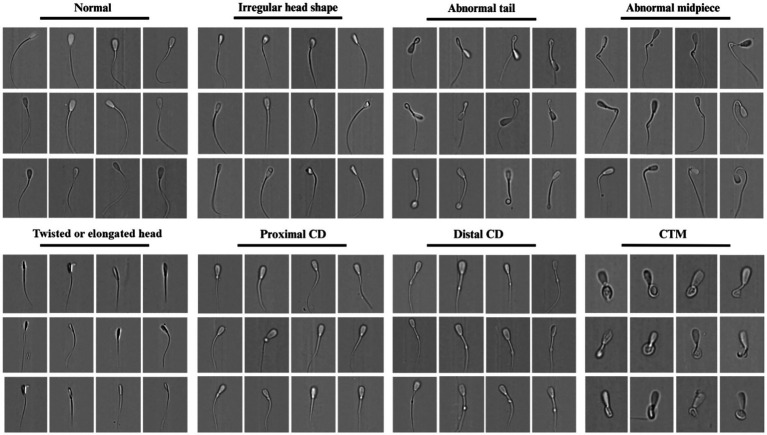
Brightfield images of bovine spermatozoa obtained from ImageStream X Mark II (Amnis-Cytek, USA) (40x magnification). Each of the eight morphological categories was detected and selected for a control set of images manually and divided into the following groups: normal, irregular head shape, twisted or elongated head, abnormal midpiece, abnormal tail, proximal cytoplasmic droplet, distal cytoplasmic droplet, and coiled tail and midpiece.

Images were manually labeled into eight morphological groups: normal morphology (NM), irregular head shape (IHS), twisted or elongated head (TEH), abnormal midpiece (AM), abnormal tail (AT), proximal cytoplasmic droplet (PCD), distal cytoplasmic droplet (DCD), and coiled tail and midpiece (CTM) (78), along with a residual (after preliminary gating) categories for multiple events and debris (together – 10 categories). To minimize overlap, we applied the following rules: (i) CTM supersedes AT when coiling of tail/midpiece is evident; (ii) TEH vs. IHS - TEH is assigned when twisted or elongated head, IHS is assigned when observing defects other than twisted or elongated head; (iii) ‘Multiple’ and ‘Debris’ were flagged for dataset hygiene and future application of models. Filtering and browsing were performed in IDEAS vs. 6.2 (Amnis-Cytek, USA). Two independent raters labeled images by manually selecting images; disagreements were resolved by consensus. We applied stratified random sampling relative to bull samples, until 200 quality labeled images per class (including “multiple” and “debris”) × breed × condition were obtained, yielding 12,000 labeled images with uniform distribution across classes, bulls/breeds, and fresh/frozen conditions (Figure 1).

### 2.5 Model training pipeline

#### 2.5.1 Data preparation

**Figure 2:**
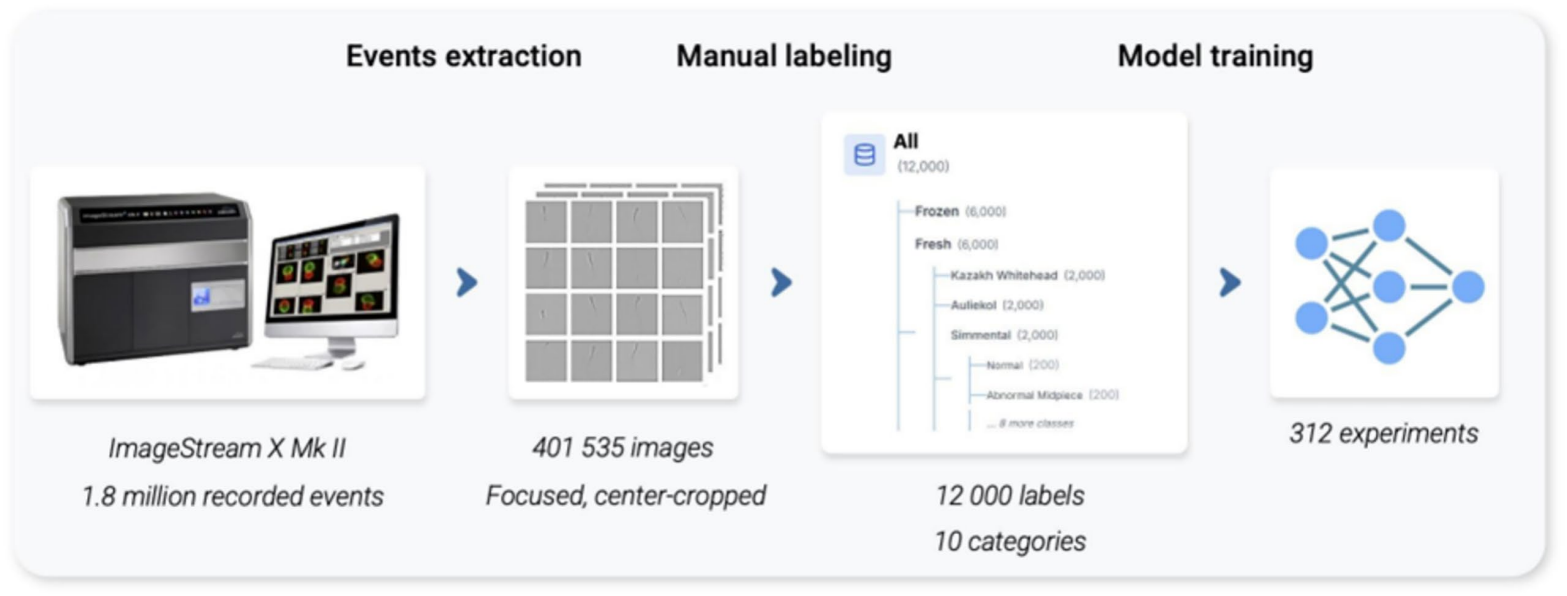
Overview of the analysis pipeline for the data selection, processing, and analysis stages. Events extraction: the images captured on ImageStreamX Mark II (Amnis-Cytek, USA) were gated to constitute focused quality images and preprocessed with center-cropping to a square ratio for uniform resizing. Manual labeling: images are labeled by two experts into ten categories (including residual debris/multiple event categories). Model training: multiple models were trained and evaluated in a series of experiments.

Brightfield images were converted to RGB by channel replication and resized to 224 × 224. Online augmentations were applied only to the training split: random horizontal flip, small rotations (±10°), and random resized crop retaining 80–100% of area with up to ±10% translation (Figure 2).

#### 2.5.2 Training and validation strategy

To ensure fair comparisons across experiments, we trained on all available labeled images using online augmentation while enforcing class-balanced sampling and per-class caps; when applicable, we also applied per-breed/per-bull caps. This approach maintained the effective exposure per class and per stratum comparable across settings and prevented data volume from acting as a confounder without discarding annotated data. The same curation was used whenever experiments were restricted to a single breed or a single condition, so that class distribution and training budget remained consistent across scenarios. We perform multiple experiments to (i) select efficient model architecture and training strategies and (ii) assess generalization of the chosen model across different conditions and breeds.

Generalization was assessed using two complementary protocols. First, to examine cross-condition transfer, we trained one model on all Frozen images (aggregated across breeds) and another on all Fresh images. Then we evaluated each model both within its training condition and across the other condition. Second, to investigate cross-breed transfer within a single condition, we trained three single-breed models per condition (Kazakh Whitehead, Simmental, Auliekol) and evaluated each model in-breed and on the two unseen breeds within the same condition. For these domain-shift analyses, we kept the same effective training budget and stratification by class (and by bull where available) to ensure fair comparisons across breeds and conditions.

For model selection, we used leave-one-breed-out (LOBO), treating the held-out breed as a test and reserving 20% of the training data for per-epoch validation. We run the same model selection procedure at different labeled-data fractions f∈{0.1, 0.2, 0.5, 1.0}. For the generalization experiments, we used fixed, stratified train/val/test = 80/10/10 splits (no cross-validation). Breakdown of label usage for all experiments is provided in Supplementary Table S3. Unless stated, results are meant over three seeds. We report 95% confidence intervals (CIs) for accuracies via a hierarchical cluster bootstrap (resampling bulls, then samples (ejaculates), then images, B = 5,000) to account for intra-bull dependence. Differences are considered significant when the CI does not include 0.

We evaluated four CNN backbones commonly used in biological imaging and computer vision (MobileNetV3-Large, EfficientNetV2-S, ResNet-50, ConvNeXt-Tiny) (79–82) under two regimes: standard line-probing (LP) and a linear probe followed by fine-tuning (LP-FT) (83–84). In LP-FT, the classifier is trained first with the backbone frozen at ImageNet-pretrained weights, followed by end-to-end fine-tuning with a reduced learning rate. Models were implemented in PyTorch with GPU acceleration (NVIDIA GeForce RTX 4080), trained with AdamW optimizer and cross-entropy loss; unless noted, batch size was 64, with learning rates of 1 × 10^−3^ for linear probing and 1 × 10^−4^ for fine-tuning. Performance was monitored on the validation split each epoch to record the best checkpoints based on validation accuracy and enable the early-stopping callback to avoid stagnating training. The F1-score, the harmonic mean of precision and recall (F1 = 2 * (Precision * Recall) / (Precision + Recall)), balanced the trade-off between false positives and false negatives; the macro average calculates the F1 for each class independently and averages them, treating all classes equally. Because evaluation splits were stratified and per-class performance was uniform, macro-F1 closely matched accuracy; therefore, accuracy is reported in Table 1, with macro-F1 provided in the Supplementary Table S4.

**Table 1:** Summary of generalization analysis results.

Train	Test	Accuracy (%) [95% CI]
Frozen condition		
Kazakh whitehead	Kazakh whitehead	74.60 [73.9–75.3]
	Simmental	66.80 [66.0–67.6]
	Auliekol	59.86 [59.0–60.7]
Simmental	Kazakh whitehead	81.67 [80.9–82.4]
	Simmental	83.49 [82.8–84.2]
	Auliekol	75.76 [74.9–76.6]
Auliekol	Kazakh whitehead	73.76 [72.9–74.6]
	Simmental	70.13 [69.2–71.0]
	Auliekol	75.75 [74.9–76.6]
Fresh condition		
Kazakh whitehead	Kazakh whitehead	71.27 [70.5–72.0]
	Simmental	68.43 [67.6–69.2]
	Auliekol	67.99 [67.2–68.8]
Simmental	Kazakh whitehead	66.51 [65.7–67.3]
	Simmental	85.55 [84.9–86.2]
	Auliekol	60.08 [59.2–60.9]
Auliekol	Kazakh whitehead	65.09 [64.2–65.9]
	Simmental	65.12 [64.3–66.0]
	Auliekol	75.03 [74.2–75.8]
Aggregated		
Frozen	Frozen	83.58 [82.9–84.2]
	Fresh	68.75 [67.9–69.6]
Fresh	Frozen	66.12 [65.3–66.9]
	Fresh	76.39 [75.7–77.1]

Accuracy given as % with 95% confidence interval (CI). The Train column demonstrates data source used for the model training stage, and data source of test data is in the Test column.

## 3 Results

### 3.1 Model selection

**Figure 3:**
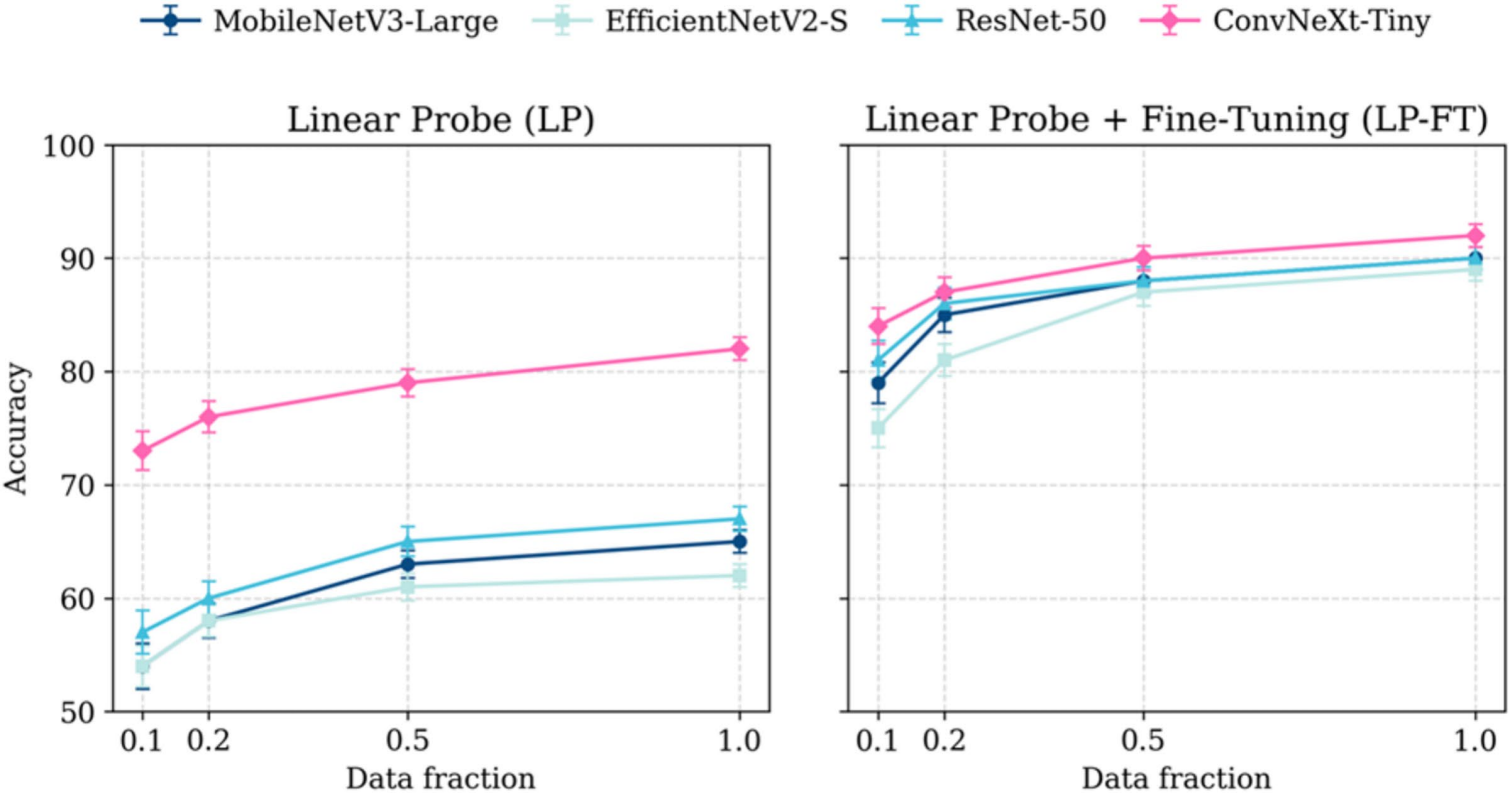
Summary of accuracy results for the tested model architectures and training strategies over four dataset fractions. Each marker shows the mean across 3 seeds per LOBO fold; error bars are ±SD across LOBO folds (k = 3).

We compared four backbones with LP and LP-FT strategies on our dataset, and repeated training at 0.1, 0.2, 0.5, and 1.0 fractions of the labeled pool to assess sample efficiency (Figure 3). For each fraction, we used the same fixed, stratified test split per seed to ensure that differences reflect training data volume rather than test drift. Under LP, accuracy increased monotonically with data, but the spread between models remained large. ConvNeXt-Tiny consistently led at every fraction, while the other backbones stayed below ~70% even at full data, reflecting underfitting and unstable convergence when only the classifier is trained. Under LP-FT, all backbones improved substantially and the gaps narrowed, yet ConvNeXt-Tiny remained best across fractions with 91.1% top accuracy. The curves show a steep gain from 0.1 to 0.2, followed by diminishing returns; by 0.5, the models approach their asymptotes, with only modest gains up to 1.0. This pattern indicates that (i) end-to-end adaptation is efficient in learning robust representations for IFC images, and (ii) ConvNeXt-Tiny is the most sample-efficient among the tested backbones. Given its consistent dominance across data regimes, strong stability under LP-FT, and favorable compute footprint, ConvNeXt-Tiny with LP-FT was selected as the primary configuration for downstream analyses.

**Figure 4:**
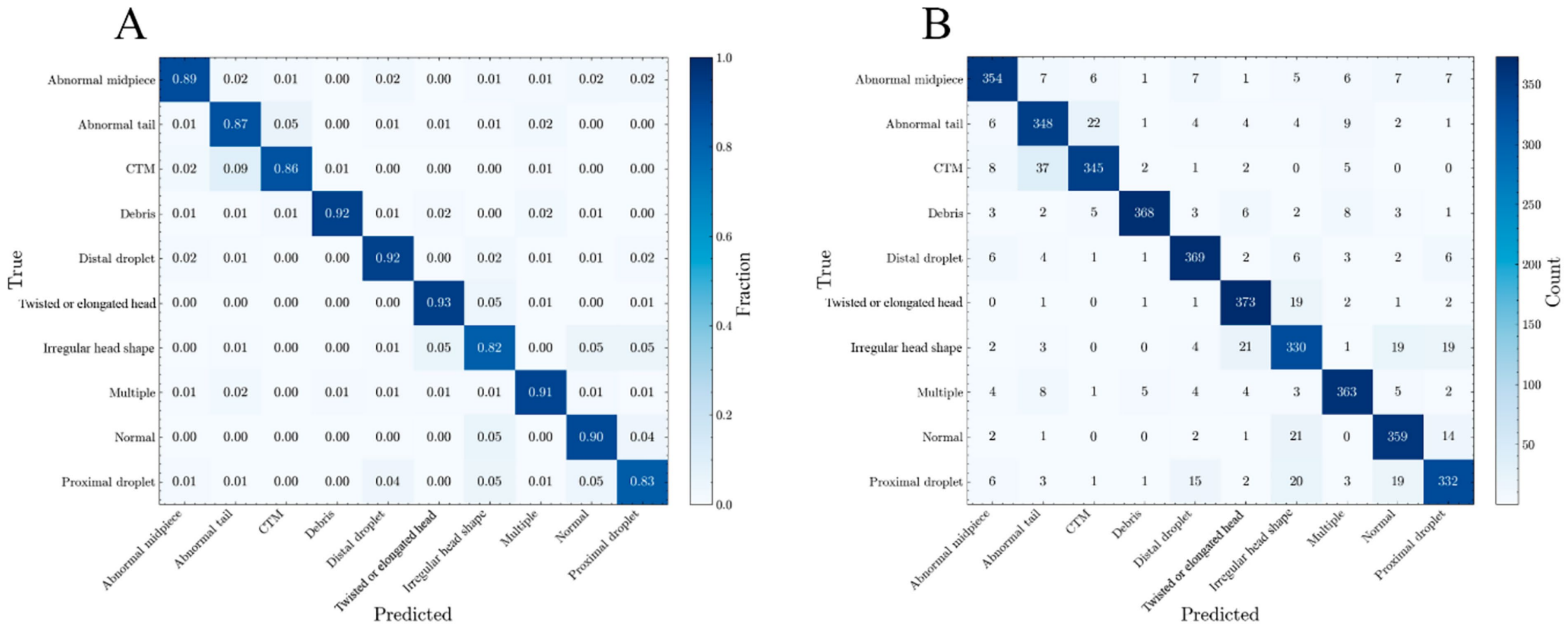
Two confusion matrices illustrate the classification performance of the ConvNeXt-Tiny model across multiple categories. **(A)** The normalized confusion matrix displays the same results as proportions (the relative classification accuracy for each category). **(B)** The non-normalized confusion matrix shows the absolute counts of predictions for each category, with rows representing the true labels and columns representing the predicted labels. Correct predictions are concentrated along the diagonal.

The analysis of the confusion matrix for the best model (Figure 4) shows strong classification accuracy across most categories. However, some confusion was noted between classes that have high visual similarity, such as CTM and AT, as well as between TEH and IHS. There was also minor confusion between the categories ‘debris’ and ‘multiple cells’. However, this does not impact biological interpretation since both categories are excluded from further analysis. Additionally, the lightweight nature of ConvNEXt-Tiny also enabled rapid inference that is suitable for high-throughput analysis.

### 3.2 Generalization analysis results

To evaluate the performance transferability across different sample preparations (frozen vs. fresh) and cattle breeds, and to assess potential biases introduced during training, we conducted a series of generalization analyses.

#### 3.2.1 Cross-condition performance

Models trained on aggregated data from a single condition performed best when evaluated under the same condition. The frozen → frozen setting reached 83.58% accuracy, and the fresh → fresh setting reached 76.39% (Table 1). When assessed across conditions, accuracy dropped by about 10–15 percentage points: frozen → fresh fell to 68.75% (−14.83 pp) and fresh → frozen to 66.12% (−10.27 pp). These results indicate a substantial data distribution and domain shift between fresh and frozen preparations that is not fully compensated by standard augmentation.

#### 3.2.2 Cross-breed performance

Within each condition, single-breed models consistently performed best on their own breed and declined on unseen breeds (Table 1). In the frozen subset, self-evaluation accuracies were 74.60% (Kazakh Whitehead), 83.49% (Simmental), and 75.75% (Auliekol). Cross-breed transfer ranged from 59.86% (Kazakh Whitehead to Auliekol) to 81.67% (Simmental to Kazakh Whitehead), showing that Simmental-trained models generalize relatively well to Kazakh Whitehead in frozen samples, whereas Auliekol, as a target, is harder for models trained on other breeds. In the fresh subset, self-evaluation reached 71.27% (Kazakh Whitehead), 85.55% (Simmental), and 75.03% (Auliekol). Cross-breed transfer was again lower, spanning 60.08% (Simmental to Auliekol) to 68.43% (Kazakh Whitehead to Simmental). Overall, cross-breed generalization typically lags self-breed performance by ~5–15 percentage points, with the largest drops observed when transferring to Auliekol. These patterns suggest breed-specific morphology and/or imaging differences coupled with sample sizes that limit zero-shot transfer from single-breed training.

### 3.3 Categorization of sperm images into morphological groups using trained CNN model

Following the completion of CNN model training and cross-validation experiments, all 401,535 images were subject to automatic labeling with the best ConvNeXt-Tiny checkpoint to perform morphological profiling. The classification results are summarized in Supplementary Table S5, with frozen and fresh samples reported separately. Additionally, the fresh semen group was further subdivided by season, encompassing spring and fall.

Head shape anomalies, particularly irregular heads shape, were abundant in both frozen and fresh groups. Morphological abnormalities like distal cytoplasmic droplets (DCD), proximal cytoplasmic droplets (PCD), and abnormal midpiece (AM) were notably present in all of the bulls across all groups. These findings underscore the combined impact of sample preservation method and seasonal variation on sperm quality, which should be carefully considered in reproductive assessments.

## 4 Discussion

The analysis of morphological abnormalities, as well as other semen parameters for assisted reproductive technologies (ART) or *in vitro* fertilization (IVF), is typically conducted using a light microscope and computer-assisted semen analysis (CASA) systems (85). While most commercial CASA systems can effectively measure sperm motility, they cannot evaluate sperm morphological abnormalities and viability. Several limitations are associated with these techniques. Firstly, they require trained personnel and experts to accurately count and categorize cells into respective morphological groups. Other limitations include a small number of images, low magnification of obtained images, the need for staining to visualize cells, an imbalance between normal and abnormal cell numbers, and increased noise in the resulting images (71).

Spermatozoa morphological abnormalities arise due to several factors, including mechanical damage, oxidative stress, incomplete cell maturation, pathological diseases, and others (13, 14, 86, 87). These abnormalities can significantly impact important sperm parameters such as plasma membrane integrity, viability, motility, mitochondrial membrane potential, DNA composition, and acrosome capacitation (16–29, 88). Therefore, categorizing these morphological abnormalities is crucial for identifying potential physiological or metabolic changes in the cells and for evaluating the overall fertilizing ability of semen. IFC has proven to be a reliable tool that facilitates robust statistical analysis for the identification of sperm morphology (49, 56–58, 60).

Deep learning algorithms allow rapid categorization of cell images into distinct morphological groups (89, 90). The IDEAS software (Amnis-Cytek, USA) already allows for significant opportunities for the analysis of images, providing focus metrics, masking, feature extraction, and rule-based classification or gating. In contrast, CNN offers end-to-end pattern learning from raw images that improves consistency and scalability. Thus, Matamaros-Volante and co-authors (91) used proprietary IDEAS software (Amnis-Cytek, USA) to analyze focused images and created masks for specific regions of spermatozoa to study capacitation-induced tyrosine phosphorylation of human spermatozoa. Several computer vision-based approaches were used for sperm quality estimation and classification of abnormal human sperm. Some of these approaches rely on automatic features (71), while others have applied more advanced feature engineering strategies alongside segmentation to gain deeper insights into a wider spectrum of morphological abnormalities (75, 92–93). Thus, Iqbal and co-authors (94) had developed a CNN optimized for the human sperm head abnormalities, successfully addressing inter-class similarity and intra-class variability.

However, only recently have suitable databases and AI algorithms have been implemented to classify boar sperm (69, 95). Keller and colleagues (69) developed machine learning-assisted classification of boar semen acquired with the IFC instrument into five different morphological groups with 20x, 40x, and 60x magnifications using Amnis-Cytek’s proprietary AI module (69). For each sample and morphological group, 2,000 images were manually labeled and used for supervised learning to evaluate morphological defects and acrosome health status. Based on the study, it was determined that more morphological categories could arise under higher magnification for label-free differentiation of the acrosome status in boar semen.

Moreover, Fraczek and co-authors (92) utilized Mask R-CNN for the automated segmentation of the sperm head and flagellum from single-channel images. However, their method exhibited low precision when segmenting the spermatozoa tail. Additionally, the size of the database used by some research groups for CNN models’ optimization was limited to 19–20 images, although it included up to 210 single-sperm events (96, 97). Hernandez-Herrera and colleagues (98) applied a ResNet-50 convolutional neural network to filter out irrelevant images and a second CNN, U-Net 2D, to segment head and midpiece images. Nonetheless, the above-mentioned research primarily focused on analyzing human spermatozoa datasets.

In our study, we analyzed a comprehensive dataset comprising 401,535 single-cell, high-resolution images of fresh and frozen spermatozoa from different Kazakhstani bull breeds (specifically, Kazakh Whitehead and Auliekol) as well as the widely distributed across Kazakhstan Simmental breed. We acquired these images using 40x magnification since other magnifications, such as 60x and 20x, would yield a smaller image dataset and lower image quality, respectively. In a series of experiments, multiple classification strategies and architectures were evaluated using consistent training and data preparation strategies, including MobileNetV3-Large, EfficientNetV2-S, ResNet-50, and ConvNeXt-Tiny; the latter was chosen as the primary model. The ConvNeXt-Tiny was able to achieve an accuracy of 91.1% on a uniformly mixed dataset containing images from various samples and breeds. Utilizing the selected training pipeline, we examined the transferability of learned features across different sample conditions and breeds. As expected, we observed slight classification confusion for morphologically overlapping classes, namely TEH and IHS, CTM and AT. Additionally, generalization worsened when the data originated from isolated collections, such as a single breed or bull, which was especially significant in the Auliekol case, despite no significant visual discrepancies. Although a fair evaluation of classification methods was attempted, it is worth noting that significantly larger dataset sizes are required to confirm biological bias in domain shift and not instrument-related or personal bias. The results of the cross-validation experiments highlighted a common conclusion: better dataset quality, variety, and size requirements are essential for differentiating true biological variability from apparent model bias due to underrepresented features.

The trained model was used to classify a large dataset of single-cell images (*n* = 401,535) into eight morphological groups (10 categories, including residual multiple events/debris). On average, approximately 58% of spermatozoa in frozen samples exhibited normal morphology, compared to 38 and 56% in fresh samples collected during the spring and fall seasons, respectively. The proportion of almost all morphologically abnormal sperm cells was reduced, though not always significantly. These data are in agreement with findings from other groups regarding the effects of cryopreservation on spermatozoa with abnormal morphology (99).

A key distinction of our approach lies in the use of IFC for sperm image training sets, which enables the rapid, high-throughput acquisition and analysis of tens of thousands of single-cell images, highlighting the gain in statistical power. On the other side, assessments under light microscopy involve a substantially smaller number of cells, typically about 100–200 cells per ejaculate, allowing for rapid evaluation but potentially limiting the detection of rare morphological defects, such as tail abnormalities or the presence of proximal cytoplasmic droplets. Furthermore, observer bias is minimized in our method, as morphological classification is performed by a CNN trained on expert-annotated data, ensuring consistent and objective evaluation across all samples.

Advantages of our approach include the analysis across eight distinct morphological classes, the use of a significantly larger database compared to previous studies, the inclusion of leading Central Asian bull breeds alongside the Simmental breed, and the comprehensive analysis of the deep learning generalization capabilities in the context of spermatozoa cell analysis.

**Figure 5:**
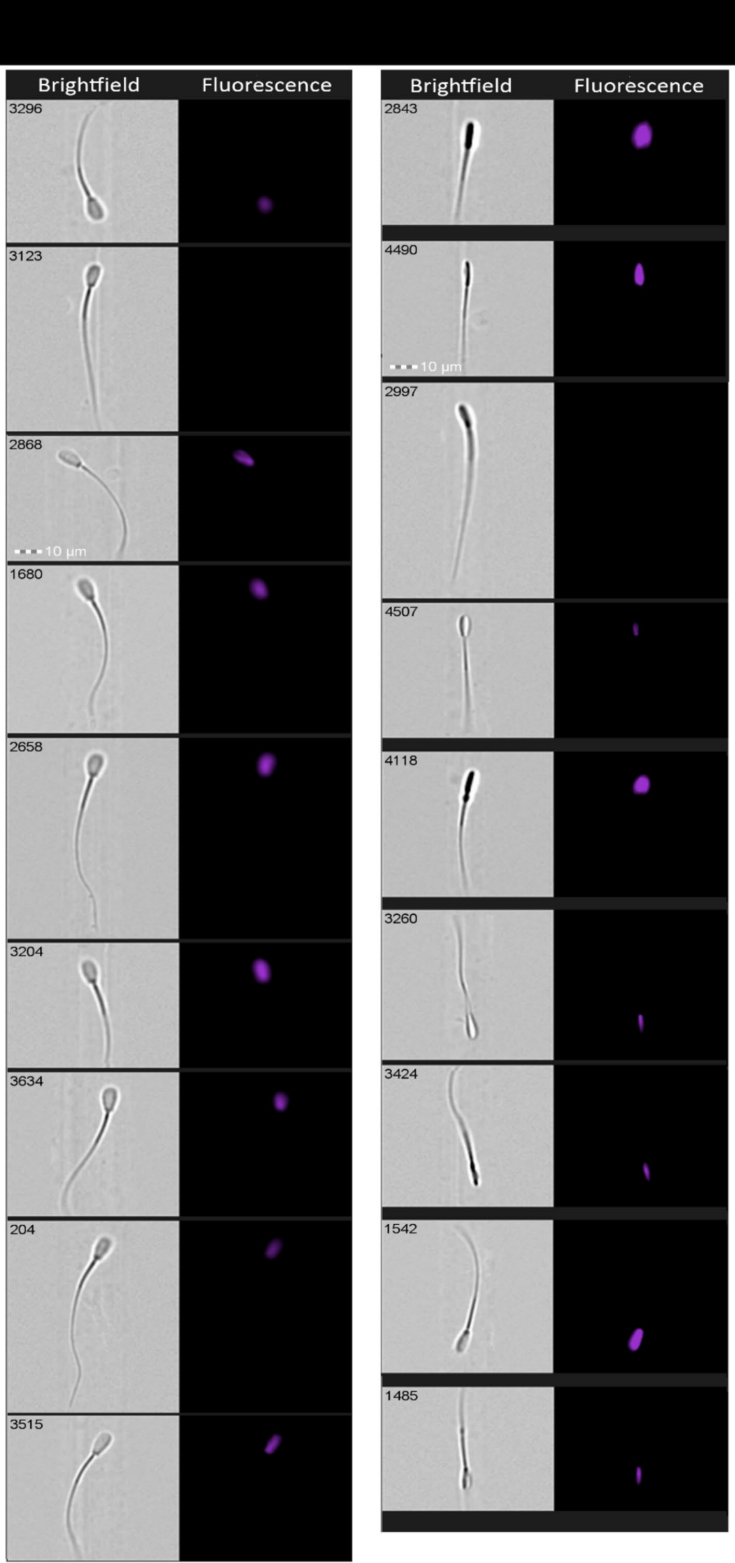
Images of spermatozoa stained with DNA dye (Hoechst 33342) and acquired with Imagestream X Mark II (40x magnification).

The main limitation of this study was the relatively low number of images in some morphologically abnormal categories due to the fact that only one ejaculate for each bull was analyzed for the fresh and frozen semen during the fall and spring seasons, and the difference between fresh sperm image quantities acquired from some bulls. The label-free analysis of asymmetric spermatozoa abnormalities may require additional second-stage verification and sperm staining to discriminate spermatozoa out of focus (Figure 5). The problem that some images of asymmetric cells from Imagestream analysis are taken slightly on angle usually solved by staining and/or additional manual verification (100). Testing of AI models that would use a combination of brightfield and fluorescence, and experimenting with crucial characteristics of the CNN architecture may enable improvements in model performance. Additional data and optimization are needed before it can be considered for monitoring morphological abnormalities in the livestock sperm.

Key areas for improvement also include enhancing the size and quality of the database used for CNN training and refinement through expanding sampling for different breeds in the future, as well as the inter-laboratory harmonization of IFC datasets obtained from different bovine breeds. There is a certain degree of intra-class similarity between visually similar categories (e.g., CTM vs. AT; TEH vs. IHS), which has been reported before for human sperm datasets (90, 99). Larger labeled datasets would provide more robust insights into potential biases in cross-validation experiments. Additionally, it is also important to note that most modern CNN and vision transformer models can effectively address classification challenges, provided there is thorough data preparation and model fine-tuning strategies. Further monitoring of data collection and preprocessing effects on the resulting performance is essential. We anticipate that advancements in AI-IFC data analysis and a thorough understanding of the cryobiology of spermatozoa will make IFC technology more accessible in the veterinary field and reduce the costs of storage and analysis.

## Data Availability

The raw data supporting the conclusions of this article will be made available by the authors, without undue reservation.

## Glossary

AI: Artificial Intelligence
AM: Abnormal Midpiece
AT: Abnormal Tail
CASA: Computer-Assisted Sperm Analysis
CI: Confidence Interval
CNN: Convolutional Neural Network
CTM: Coiled Tail and Midpiece
DCD: Distal Cytoplasmic Droplet
FC: Flow cytometry
IFC: Imaging Flow Cytometer
IHS: Irregular Head Shape
IVF: *In Vitro* Fertilization
LOBO: Leave-One-Breed-Out
LP-FT: Linear Probing and Fine Tuning
MMP: Mitochondrial Membrane Potential
NM: Normal Morphology
PCD: Proximal Cytoplasmic Droplet
PI: Propidium Iodide
PM: Progressive Motility
TEH: Twisted or Elongated Head
TM: Total Motility
VAP: Average Path Velocity
