# Supplementary material for: Deep learning-enabled morphology analysis of bovine sperm for label-free imaging flow cytometry
Source: Front Vet Sci. 2026 May 8;13:1634224. doi: 10.3389/fvets.2026.1634224 (PMC13193903; doi:10.3389/fvets.2026.1634224)
Supplement: Supplementary file 1 [file Data_Sheet_1.docx]

**Deep Learning-enabled Sperm Morphology Analysis of Bovine Sperm for label-free Imaging Flow Cytometry**

**Supplementary tables**

**Table S1.** Photometric and kinematic data for fresh semen obtained in spring and fall seasons and frozen samples cryopreserved in summer for all 6 bulls.

| **Fresh, Spring** | | | | | | | | | | | | | | |
| --- | --- | --- | --- | --- | --- | --- | --- | --- | --- | --- | --- | --- | --- | --- |
| **Bull ID** | **Date** | **Cell conc, 10^9^/ml** | **Cell count** | **TM, %** | **PM, %** | **VAP, μm/s** | **VSL um/s** | **VCL, μm/s** | **ALH, μm** | **BCF, Hz** | **STR, %** | **LIN, %** | **Elong, %** | **Area, μm^2^** |
| Bull 1 | 23.05.2024 | - | 39 | 33,33 | 15,38 | 120.6±34.9 | 78.6±34.4 | 218.9±49.5 | 7.7±1.2 | 22.1±12.7 | 66±21 | 37±14 | 49±8.3 | 4.5±0.7 |
| Bull 2 | 04.06.2024 | 189,0 | 12 | 25,00 | 16,67 | 90.4±15.6 | 65.8±5.1 | 152±19 | 5.2±0.4 | 22.1±16 | 74±9 | 44±5 | 47±11.1 | 4±0.3 |
| Bull 3 | 04.06.2024 | 889,4 | 77 | 1,29 | 1,29 | 88.3±0 | 82.3±0 | 117.7±0 | 3.7±0 | 32.7±0 | 93±0 | 70±0 | 58±0 | 3.6±0 |
| Bull 4 | 04.06.2024 | 1473,0 | 76 | 56,58 | 43,42 | 82.9±36.5 | 73.6±35 | 137.4±44.3 | 6.6±2.4 | 24±10.6 | 89±8 | 53±12 | 43±8.9 | 5±1.2 |
| Bull 5 | 23.05.2024 | - | 38 | 60,53 | 44,74 | 141.3±28.9 | 114.7±33.8 | 221.3±47.3 | 7.4±3.4 | 26.7±20 | 81±17 | 53±14 | 49±11.5 | 4.3±0.3 |
| Bull 6 | 04.06.2024 | 863,5 | 45 | 35,55 | 17,77 | 85.5±54.6 | 55.2±33.8 | 139.7±60.9 | 4.8±0.7 | 31±10.2 | 70±21 | 41±116 | 47±11.5 | 4.9±0.8 |
| **Fresh, Fall** | | | | | | | | | | | | | | |
| Bull 1 | 10.09.2024 | 0,960 | 111 | 60,36 | 50,45 | 172.4±57 | 147.6±54.8 | 253.9±74.8 | 8.7±3 | 15.9±19.8 | 85±15 | 59±17 | 46±11.8 | 4.7±0.8 |
| Bull 2 | 06.09.2024 | 0,672 | 62 | 70,97 | 67,74 | 160.7±32.9 | 151.4±38.3 | 222.7±51 | 7.2±2.2 | 22.6±18.7 | 93±11 | 69±17 | 43±10.9 | 4.8±0.8 |
| Bull 3 | 06.09.2024 | 1,092 | 273 | 71,79 | 41,39 | 65.9±35.4 | 63.5±34.1 | 76.9±50.2 | 2.7±2.5 | 29.9±12.5 | 97±7 | 88±15 | 47±13.3 | 5.5±5 |
| Bull 4 | 06.09.2024 | 1,373 | 219 | 80,82 | 70,32 | 158.5±44.7 | 138.6±48.3 | 236.6±61 | 8.6±3.4 | 18.7±17.7 | 87±15 | 60±19 | 43±11.1 | 4.8±1.4 |
| Bull 5 | 10.09.2024 | 1,015 | 134 | 90,30 | 76,12 | 152.7±54.3 | 134.9±56.8 | 214.2±71.7 | 7.5±2.8 | 18.3±18.6 | 88±15 | 65±20 | 42±13.1 | 5.5±8.1 |
| Bull 6 | 06.09.2024 | 0,512 | 103 | 70,87 | 54,37 | 136.2±47.6 | 116/1±48.2 | 197.6±69 | 7.6±2.9 | 22.7±18.9 | 84±15 | 59±16 | 46±14.4 | 4.7±1.1 |
| **Frozen, Summer** | | | | | | | | | | | | | | |
| Bull | Date frozen | Date thawed | Cell count | TM, % | PM, % | VAP, μm/s | VSL μm/s | VCL, μm/s | ALH, μm | BCF, Hz | STR, % | LIN, % | Elong, % | Area, μm^2^ |
| Bull 1 | 02.05.2024 | 10.09.2024 | n/a | n/a | n/a | n/a | n/a | n/a | n/a | n/a | n/a | n/a | n/a | n/a |
| Bull 2 | 06.08.2024 | 06.09.2024 | 671 | 84.2 | 37.7 | 62.5±39.1 | 54.3±32.4 | 83.3±62.7 | 3.9±3.5 | 20.9±13.6 | 90±16 | 75±23 | 47±15.8 | 7.6±7.6 |
| Bull 3 | 10.09.2024 | 13.09.2024 | n/a | n/a | n/a | n/a | n/a | n/a | n/a | n/a | n/a | n/a | n/a | n/a |
| Bull 4 | 27.08.2024 | 06.09.2024 | 514 | 70.23 | 44.16 | 117.3±54.8 | 92.1±56 | 176.1±76.5 | 6.4±3.8 | 12.5±16 | 78±20 | 56±23 | 44±13.3 | 4.6±1.6 |
| Bull 5 | 26.08.2024 | 10.09.2024 | 1038 | 82.85 | 45.57 | 96±54.4 | 73.3±50.4 | 149.9±76.4 | 6.9±4.2 | 17±16.1 | 76±20 | 52±23 | 45±15 | 5.6±4.2 |
| Bull 6 | 20.08.2024 | 06.09.2024 | 450 | 92.44 | 66.67 | 86.8±38.8 | 74±36.4 | 122.2±56.5 | 4.9±3.1 | 18.3±15.1 | 85±17 | 65±24 | 47±12.9 | 4.7±1.8 |

*Note*: TM - total motility, PM - progressive motility, VAP - average path velocity, VSL - straight line velocity, VCL - curvilinear velocity, ALH - amplitude of lateral head, BCF - beat cross frequency, STR - straightness, LIN - linearity, Elong - elongation, Area - area of a sperm head

**Table S2.** Descriptive data of the number of IFC images extracted from each bull sample for morphological assessment and viability analysis (before additional filtering out)

| **Bull ID** | **Fresh, Spring** | **Fresh, Fall** | **Frozen, Summer** |
| --- | --- | --- | --- |
| Bull 1 | 22614 | 18271 | 30592 |
| Bull 2 | 25595 | 22008 | 31196 |
| Bull 3 | 24267 | 23057 | 22387 |
| Bull 4 | 31457 | 19562 | 22864 |
| Bull 5 | 21905 | 20593 | 26251 |
| Bull 6 | 26568 | 20118 | 27069 |

**Table S3.** Breakdown of split sizes of the labeled data for different experimental stages.

| **Experiment** | **Total Available** | **Train** | **Val** | **Test** | **Notes** |
| --- | --- | --- | --- | --- | --- |
| Single-breed, single-condition | 1 breed × 1 condition × 10 classes × 200 = 2,000 | 1,600 | 200 | 200 | Used for within-breed self and cross-breed external tests (external tests use the target breed’s 200-image test split in the same condition). |
| Condition-aggregated (Frozen) | 3 breeds × 10 × 200 = 6,000 | 1,600 (capped) | 200 (capped) | 600 | Train on Frozen; report Frozen→Frozen (self) and Frozen→Fresh (external). |
| Condition-aggregated (Fresh) | 3 breeds × 10 × 200 = 6,000 | 1,600 (capped) | 200 (capped) | 600 | Train on Fresh; report Fresh→Fresh (self) and Fresh→Frozen (external). |
| LOBO (per fold) | Train on 2 breeds × 2 conditions × 10 × 200 = 8,000; test on held-out breed (2 cond × 10 × 200 = 4,000) | 6,400 | 1,600 | 4,000 | k=3 folds (held-out breed rotates). 3 seeds per fold. Validation is a 20% hold-out from the training pool. |
| Data-fraction runs (LP/LP-FT, per LOBO fold) | same as LOBO training pool above (8,000 total, 6,400 train after 20% val) | f × 6,400 | 1,600 | 4,000 | Fractions f∈{0.1,0.2,0.5,1.0} applied to the training split only; val/test kept fixed. So train counts are 640, 1,280, 3,200, 6,400 respectively. |

**Table S4.** Summary of generalization analysis results. Data source specified in the Train column was used for model training, and data source specified in the Test column was used for the model evaluation. Accuracy and macro F1 are reported with 95% CI.

| **Train** | **Test** | **Accuracy (%) [95% CI]** | **Macro F1 [95% CI]** |
| --- | --- | --- | --- |
| Frozen Condition | | | |
| Kazakh Whitehead | Kazakh Whitehead | 74.60 [73.9-75.3] | 0.74 [0.73-0.75] |
|  | Simmental | 66.80 [66.0-67.6] | 0.67 [0.66-0.68] |
|  | Auliekol | 59.86 [59.0-60.7] | 0.60 [0.59-0.61] |
| Simmental | Kazakh Whitehead | 81.67 [80.9-82.4] | 0.82 [0.81-0.83] |
|  | Simmental | 83.49 [82.8-84.2] | 0.83 [0.82-0.84] |
|  | Auliekol | 75.76 [74.9-76.6] | 0.76 [0.75-0.77] |
| Auliekol | Kazakh Whitehead | 73.76 [72.9-74.6] | 0.73 [0.72-0.74] |
|  | Simmental | 70.13 [69.2-71.0] | 0.69 [0.68-0.70] |
|  | Auliekol | 75.75 [74.9-76.6] | 0.75 [0.74-0.76] |
| Fresh Condition | | | |
| Kazakh Whitehead | Kazakh Whitehead | 71.27 [70.5-72.0] | 0.71 [0.70-0.72] |
|  | Simmental | 68.43 [67.6-69.2] | 0.68 [0.67-0.69] |
|  | Auliekol | 67.99 [67.2-68.8] | 0.68 [0.67-0.69] |
| Simmental | Kazakh Whitehead | 66.51 [65.7-67.3] | 0.67 [0.66-0.68] |
|  | Simmental | 85.55 [84.9-86.2] | 0.85 [0.84-0.86] |
|  | Auliekol | 60.08 [59.2-60.9] | 0.60 [0.59-0.61] |
| Auliekol | Kazakh Whitehead | 65.09 [64.2-65.9] | 0.65 [0.64-0.66] |
|  | Simmental | 65.12 [64.3-66.0] | 0.65 [0.64-0.66] |
|  | Auliekol | 75.03 [74.2-75.8] | 0.75 [0.74-0.76] |
| Aggregated | | | |
| Frozen | Frozen | 83.58 [82.9-84.2] | 0.83 [0.82-0.84] |
|  | Fresh | 68.75 [67.9-69.6] | 0.68 [0.67-0.69] |
| Fresh | Frozen | 66.12 [65.3-66.9] | 0.66 [0.65-0.67] |
|  | Fresh | 76.39 [75.7-77.1] | 0.76 [0.75-0.77] |

**Table S5.** Number of images sorted into 10 morphological groups for all the six bulls both for the frozen and fresh, conditioned by season.

| **Frozen, Summer** | | | | | | |
| --- | --- | --- | --- | --- | --- | --- |
| **Class** | **Bull 1** | **Bull 2** | **Bull 3** | **Bull 4** | **Bull 5** | **Bull 6** |
| AM | 278 | 473 | 336 | 1200 | 936 | 1159 |
| AT | 157 | 637 | 270 | 2137 | 1135 | 993 |
| CTM | 129 | 171 | 71 | 317 | 275 | 166 |
| DCD | 389 | 1849 | 113 | 187 | 395 | 540 |
| TEH | 2269 | 2736 | 2464 | 2216 | 3098 | 3572 |
| IHS | 1974 | 2011 | 1719 | 1960 | 2814 | 2895 |
| PCD | 131 | 55 | 27 | 83 | 124 | 89 |
| Multiple | 99 | 119 | 129 | 77 | 110 | 87 |
| Debris | 4025 | 8031 | 6855 | 5259 | 6736 | 5839 |
| Normal | 12662 | 12860 | 9117 | 7926 | 9181 | 10029 |
| **Total*** | 22113 | 28942 | 21101 | 21362 | 24804 | 25369 |
| **% Normal**** | 70.02 | 61.50 | 63.99 | 49.46 | 51.12 | 51.58 |
| **Fresh, Spring** | | | | | | |
| AM | 2637 | 611 | 137 | 89 | 1419 | 1400 |
| AT | 982 | 360 | 65 | 179 | 773 | 2174 |
| CTM | 439 | 436 | 46 | 197 | 466 | 868 |
| DCD | 469 | 705 | 257 | 604 | 936 | 1361 |
| TEH | 2512 | 3267 | 3313 | 3208 | 3173 | 2822 |
| IHS | 2761 | 1807 | 1525 | 2007 | 2029 | 1485 |
| PCD | 3023 | 3512 | 3712 | 5217 | 2385 | 1755 |
| Multiple | 211 | 441 | 695 | 725 | 210 | 402 |
| Debris | 4918 | 4658 | 3771 | 4815 | 5565 | 3939 |
| Normal | 3359 | 8519 | 9556 | 11268 | 4052 | 7837 |
| **Total*** | 21311 | 24316 | 23077 | 28309 | 21008 | 24043 |
| **% Normal**** | 20.49 | 44.33 | 51.35 | 49.49 | 26.24 | 38.98 |
| **Fresh, Fall** | | | | | | |
| AM | 1520 | 1209 | 115 | 826 | 316 | 1489 |
| AT | 751 | 683 | 78 | 109 | 190 | 738 |
| CTM | 203 | 92 | 26 | 28 | 45 | 51 |
| DCD | 356 | 163 | 93 | 135 | 217 | 380 |
| TEH | 661 | 396 | 1731 | 318 | 366 | 384 |
| IHS | 777 | 1522 | 1802 | 1704 | 2135 | 1384 |
| PCD | 539 | 240 | 88 | 288 | 216 | 213 |
| Multiple | 287 | 560 | 270 | 490 | 345 | 389 |
| Debris | 7525 | 9698 | 6629 | 8623 | 11083 | 8760 |
| Normal | 4326 | 5948 | 10787 | 5884 | 4965 | 4634 |
| **Total*** | 16945 | 20511 | 21619 | 18405 | 19878 | 18422 |
| **% Normal**** | 45.92 | 55.01 | 71.96 | 60.15 | 56.45 | 47.96 |

*Note:* *Total number of images was calculated by excluding non-suitable images (as described in Section 2.5.1) from the total number of acquired images, as shown in Table S2.

**The percentage of cells with normal morphology was calculated by dividing the number of cells classified as "normal" by the total number of analyzed cells, excluding events labeled as “Multiple” and “Debris.”
